# Successful Treatment With Mepolizumab for Eosinophilic Granulomatosis With Polyangiitis: A Case Report

**DOI:** 10.7759/cureus.38797

**Published:** 2023-05-09

**Authors:** Andrés Toscano Peña, Abraham Ali Munive, Yaicith Arevalo

**Affiliations:** 1 Pulmonology, Universidad De La Sabana, Bogotá, COL; 2 Pulmonology, Fundación Neumológica Colombiana, Bogota, COL; 3 Pulmonology, Fundación Neumológica Colombiana, Bogotá, COL; 4 Allergy and Immunology, Fundación Neumológica Colombiana, Bogotá, COL

**Keywords:** anca- associated vasculitis, mepolizumab, severe asthma, eosinophilic granulomatosis with polyangiitis, churg strauss syndrome

## Abstract

Eosinophilic granulomatosis with polyangiitis (EGPA) is an uncommon antineutrophil cytoplasmatic antibody (ANCA) associated vasculitis involving small and medium size blood vessels. It has a variable clinical presentation depending on the main organ involved, making it difficult to diagnose. Treatment is mainly based on high-dose steroids and other immunosuppressants like cyclophosphamide, which may prevent end-organ damage and induce remission at the expense of having important adverse effects. However, new therapeutic agents had been shown to provide better results with favorable safety profiles. Biologic therapy with monoclonal antibodies such as Rituximab and Mepolizumab has been approved for its use in ANCA vasculitis including eosinophilic granulomatosis with polyangiitis.

These cases describe two patients with EGPA whose initial presentation was severe asthma and who appeared to have extrapulmonary end-organ damage. Mepolizumab was used in both cases with a successful response.

## Introduction

Eosinophilic granulomatosis with polyangiitis (previously known as Churg-Strauss syndrome) is an antineutrophil cytoplasmic antibody (ANCA) mediated vasculitis characterized by the presence of asthma, small and medium size vessel necrotizing vasculitis, rhino-sinusitis, and tissular and peripheral eosinophilia [1]. Its prevalence in the general population is 10.7 per million and it has an annual incidence of 1 to 4/million/year. The incidence in the asthmatic population goes from 34.6 to 64.4 million/year. Before 1950, the survival rate among patients with ANCA vasculitis was five months. However, after the implementation of steroids and alkylating agents, like cyclophosphamide (CYC) in the 70’s decade, survival rates increased up to 80% to 10 years. Treatment with glucocorticoids and cyclophosphamide had been the standard management for ANCA vasculitis including eosinophilic granulomatosis with polyangiitis until 2010 when the non-inferiority of Rituximab vs cyclophosphamide was demonstrated in patients with end-organ damage [2].

Cyclophosphamide´s adverse effects such as the risk of cancer in young patients and gonadal toxicity in women of childbearing age remain the most important limiting factor and an uncertainty issue when establishing the suitable time and dose of treatment in vasculitis. Taking this into account, the focus of EGPA treatment in recent years has been the role eosinophils play in the physiopathology of this disease. As IL-5 is the strongest eosinophil activator, the use of anti-IL-5 monoclonal antibodies in the treatment of EGPA has been evaluated given the fact that this molecule has proven efficacy for the treatment of severe eosinophilic asthma. Randomized clinical trials have demonstrated the efficacy of anti-IL-5 monoclonal antibodies in the treatment of EGPA. Mepolizumab was the first anti-IL-5 monoclonal antibody approved by the FDA and EMA for the treatment of this vasculitis in 2017 [2, 3].

In this article, we present the cases of two patients with eosinophilic granulomatosis with polyangiitis in whom the treatment with Mepolizumab was effective.

## Case presentation

Case 1

**Figure 1:**
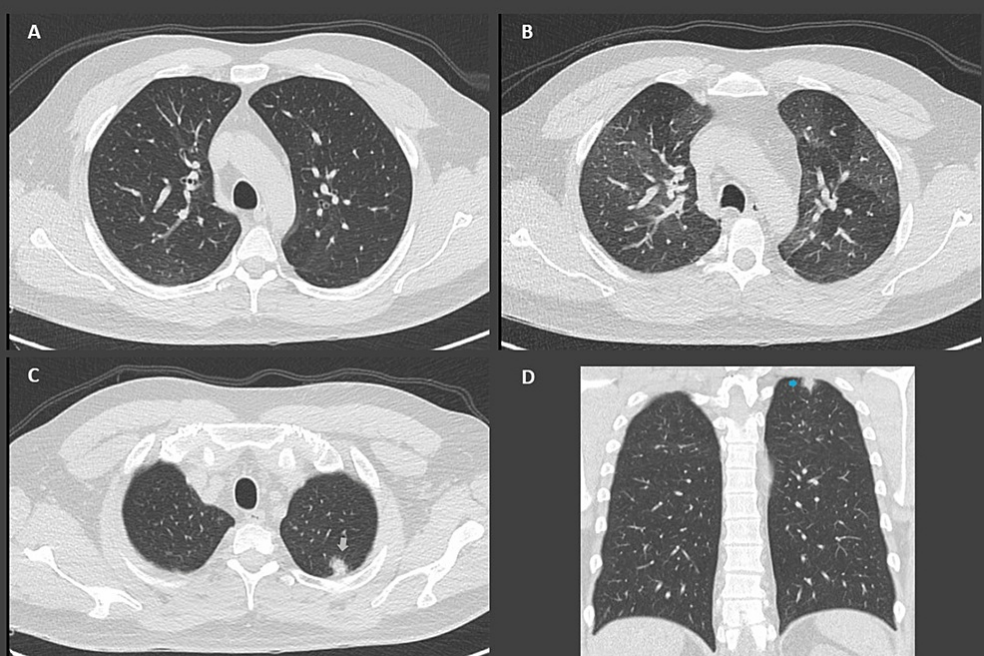
CT scan for Patient 1 A: The CT scan shows lung slides during the inspiratory phase. B: shows mosaic attenuation pattern on expiratory phase slides. C-D: A nodule in the apico-posterior segment of the left upper lobe (yellow arrow) surrounded by a ground-glass zone (blue arrow)

The patient is a 40-year-old male with a history of chronic cough, dyspnea, wheezing, and chest tightness. A mild airflow obstruction (FEV1/CVF:< 70% and FEV1: 88%) with significant reversibility after the administration of inhaled bronchodilator was detected by spirometry, which confirmed asthma. Treatment with an intermediate dose of combined inhaled corticosteroid (ICS) and long-acting beta agonist (LABA) was initiated. During follow-up, laboratory tests for biomarkers showed peripheral eosinophilia with a maximum count of 1,267 10^3^/μl (13.9%). A paranasal computed tomography (CT) scan showed chronic pansinusitis and a thorax CT scan showed a nodule in the left upper lobe with a halo sign and subsegmental air trapping zones as expiratory images were obtained (Figure 1). Vasculitis was among the differential diagnosis after ruling out infectious diseases, and his condition was classified as EGPA according to 1990 ACR classificatory criteria.

Oral glucocorticoids were given as a first-line treatment and symptoms were partially relieved. On follow-up, there was a decline in FEV1 to 59%. The patient experienced adverse effects to steroids such as weight gain and prediabetes, due to which glucocorticoids were tapered, and subsequently, anti-IL-5 biologic therapy with Mepolizumab 100 mg every four weeks was started.

The patient had a satisfactory course with an improvement of respiratory symptoms and a drop in eosinophil count to 0X10^3^/μl. Azathioprine was prescribed as maintenance therapy and there was no decline in pulmonary function as his last FEV1 was 3.11 L (81%).

Case 2

The patient is a 68-year-old female with a history of asthma since 2015 reported increasing dyspnea, cough, and nocturnal wheezing after an aortic valve replacement procedure. She received treatment with Budesonide 80 mcg/Formoterol 4.5 mcg every 12 hours at the time. Spirometry showed an obstructive pattern with FEV1 730 ml (31%) and significant reversibility after the administration of an inhaled bronchodilator. 

**Figure 2:**
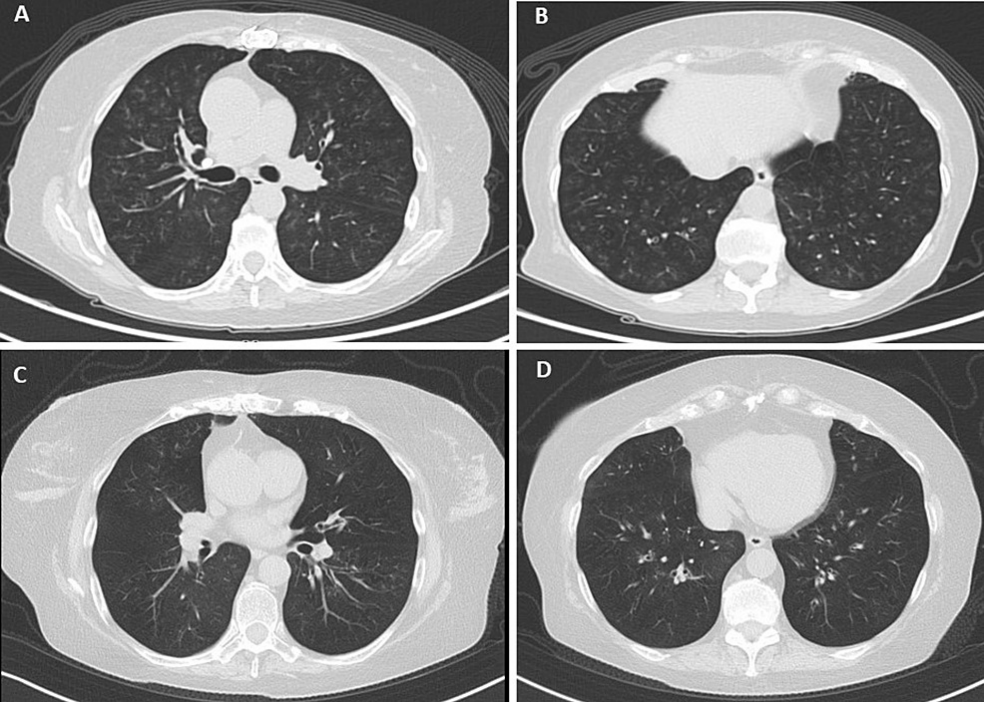
CT scan for Patient 2 A-B: The CT scan shows centrilobular ground glass nodules with tree-in-bud pattern in the upper and lower lobes of both lungs. C-D: Control CT scan shows improvement in parenchymal compromise after mepolizumab was started. The ground glass centrilobular nodules disappeared.

During follow-up, the patient was not able to achieve adequate symptom control and had a persistent severe airflow limitation according to the follow-up spirometry despite the aforementioned treatment; therefore the ICS-LABA dose was adjusted to Budesonide 320 mcg/Formoterol 9 mcg. A chest CT scan showed ground glass opacities and centrilobular nodules with a tree-in-bud distribution pattern (Figure 2A, 2B). Treatment with combined inhaled ICS-LABA was maintained with clinical stability.

Two years later, she was admitted to the emergency room with a fever and shortness of breath. Laboratory tests confirmed the presence of acute on chronic kidney disease with hematuria and non-nephrotic proteinuria supported by a 24-hour urine protein test that showed 890.4 mg protein in 1680 ml of urine volume. The complete blood count had an absolute eosinophil count of 640 cells. Due to suspicion for a systemic disease like vasculitis, an autoimmune panel was performed. The initial autoimmune biomarkers: antinuclear antibodies (ANA), anti-DNA, rheumatoid factor, anti-SCL70, and anti-JO1 were negative, and C3 and C4 complement were normal. However, antineutrophil cytoplasmatic antibodies were positive with a p-ANCA pattern of 1/640. A renal biopsy showed advanced-stage chronic nephropathy with global and focal-segmental glomerulosclerosis compatible with segmental necrotizing glomerulonephritis. Given these findings, the diagnosis of eosinophilic granulomatosis with polyangiitis was made.

Induction therapy with Rituximab was started and subsequent maintenance treatment with prednisone and azathioprine was indicated. During the follow-up she had a decline in pulmonary function tests and quality of life, due to which anti IL-5 biologic therapy was started with Mepolizumab at a dose of 100 mg every four weeks. She had clinical stability without an asthma crisis, there was an improvement in lung parenchymal compromise in the CT scan (Figure 2C, 2D), and kidney function remained stable. The eosinophil count was lowered to 0 cells and a Birmingham Vasculitis Activity Score (BVAS) of 0 was maintained during the follow-up.

## Discussion

Glucocorticoids had been the cornerstone in the treatment of eosinophilic granulomatosis with polyangiitis due to their effect on blood and tissue eosinophil count reduction, through the induction of apoptosis and the inhibition of cell survival pathways [4]. Although 90% of patients achieve remission criteria, almost 25% have a disease relapse, and 18% present asthma crisis and rhino-sinusitis symptoms exacerbation [5]. High-dose steroid schemes are used during long periods of time, increasing the likelihood of producing adverse effects including risk of infections, diabetes, adrenal insufficiency, and osteoporosis [6].

The addition of other cytotoxic immunosuppressants like cyclophosphamide in the treatment of severe EGPA has been justified with extrapolated data from trials made for other kinds of ANCA vasculitis. Without maintenance treatment, relapse rates range between 73.8 and 85.7% [7]. Given the adverse effects produced by the use of glucocorticoids for long periods and other immunosuppressants used in EGPA such as azathioprine or methotrexate, there has been more interest in investigating alternative therapies with mechanisms of action focused on anti-inflammatory mediators like IL-5, which is involved in eosinophil differentiation, maturation and propagation.

Mepolizumab is a humanized monoclonal antibody that binds to free circulating IL-5 with a high affinity and specificity managing to block the interaction between the IL-5 receptor $\alpha$-chain localized in the eosinophil cell surface. Mepolizumab use was initially approved for the treatment of severe eosinophilic asthma at a dose of 100 mg every four weeks [8]. Later, the MIRRA trial results were published, which evaluated refractory EGPA patients receiving 7.5 mg a day of corticosteroids. The study population underwent randomization and was assigned into two groups to receive Mepolizumab 300 mg every four weeks vs placebo. The results showed more accrued weeks of remission in the Mepolizumab group (28% vs 3% of patients had > 24 weeks of remission, p < 0.001) with minimum adverse effects [9]. In a posthoc analysis, an additional clinical benefit in the reduction of steroid dose and a lower rate of relapse was observed with the use of Mepolizumab [10].

Mepolizumab efficacy at a dose of 100 mg every four weeks has been evaluated in recent trials. A collaborative European trial evaluated the use of biologic therapy in refractory EGPA and found that 100 mg of Mepolizumab had a better effect in glucocorticoid tapering for steroid-dependent patients with asthmatic phenotype compared to Omalizumab [11]. The latest work is a multicenter observational trial carried out in Europe, which showed that in patients with EGPA, Mepolizumab was effective in the reduction of the Birmingham Vasculitis Activity Score (BVAS), the prednisone dose, and the blood eosinophil count up to a 24 month period without significant differences between 100 mg every four weeks and 300 mg every four weeks [12].

In patient 1, ACR criteria were used to classify as EGPA. Although ANCA was negative, there are descriptions of seronegative variants of the disease that could profit from immunosuppressive treatment with good response [5]. Biologic therapy was added due to refractory asthma with persistent eosinophilia, rhino-sinusitis, and the adverse effects developed during the treatment with glucocorticoids. 

Mepolizumab was chosen over methotrexate, mycophenolate, or azathioprine according to the ACR guidelines recommendation for patients with relapse consisting of non-severe manifestations of the disease such as asthma or IgE levels [13]. Additionally, clinical stability was achieved with a Five factor score (FFS) of 0 points as a good prognostic marker.

In case 2, respiratory symptoms and the diagnosis of asthma were the landmarks of the disease. However, extrapulmonary compromise with pauci-immune focal glomerulosclerosis detected by renal biopsy was important in narrowing the differential diagnosis and classifying the patient as EGPA. Induction treatment with Rituximab was based on the benefit over cyclophosphamide in severe disease demonstrated in clinical trials [13]. As she had refractory asthma despite the treatment and having into account the extrapulmonary manifestations of the disease, mepolizumab was added. The effective response in this patient can be explained by several factors, including a low BVAS before treatment and a high blood eosinophil count which is a good response predictor. The patient has good prognostic factors like an FFS in 1 point including the new items added in the 2011 update which include age > 65 and rhino-sinusitis [14].

## Conclusions

Eosinophilic granulomatosis with polyangiitis (EGPA) treatment represents a challenge among physicians. Understanding the pathophysiologic mechanisms involved in the development of the disease has allowed alternative treatment options with different mechanisms of action and impact in the inflammatory cascade to emerge. Understanding the role of eosinophils and their mediators has supported the use of Mepolizumab as the only anti-IL5 approved agent for the treatment of EGPA given its efficacy and safety profile in clinical trials. Mortality reduction with minimum adverse effects with Mepolizumab represents significant progress in the treatment of ANCA vasculitis. However, further research is required to define the adequate starting time and the optimal duration of treatment with these drugs.
